# Clean energy benefits the climate, the economy and our health

**DOI:** 10.2471/BLT.16.030716

**Published:** 2016-07-01

## Abstract

Jeffrey D Sachs tells Fiona Fleck why investing in renewable energy is good for our health, but why poor countries need more time to make the switch.

Q: At the Second Global Conference on Health and Climate in Paris (7–8 July) you will be asked to present on the WHO Working Group on Health Economics and Climate Change. Can you tell us what the new group will be doing?

A: The new working group will help to organize our scientific and economic knowledge related to health economics and climate change in three areas. One, the links between climate change, fossil fuel use and public health; two, the economic costs of the disease burden due to climate change and fossil-fuel use; and three, the co-benefits of decarbonization for health and climate safety. This working group will conduct several consultations with key health and economics experts around the world – including many virtual ones – during 2016–7 to collect information and examine local contexts. It will hopefully report to the World Health Assembly in May 2017.

Q: We know from the Intergovernmental Panel on Climate Change reports – the largest collection of scientific evidence on climate change – that climate change has important economic implications. But how do these economic implications relate to health?

A: Global warming is altering ecosystems and human settlements in several ways that threaten human health and well-being. Extreme weather events – including droughts, floods, heat waves and extreme storms – directly claim lives and destroy livelihoods. The changing climate destabilizes food production, and can lead to hunger and even famine. Warmer temperatures are extending the range of various tropical disease vectors, such as those for malaria, dengue fever and Zika virus. As human and animal habitats change because of global warming, emerging and re-emerging diseases such as Ebola, MERS (Middle East respiratory syndrome), SARS (severe acute respiratory syndrome) and other zoonotic (animal diseases transmitted to humans) diseases are likely to appear. Global warming is also likely to contribute to more stagnant air masses, especially in the tropics and subtropics, thereby contributing to higher levels of air pollution from soot, tropospheric ozone, and other causes, and air pollution is now recognized as the leading environmental cause of death globally.

Q: Countries agreed to limit global warming at the United Nations climate change conference in Paris last year. About 80% of the world’s energy is from carbon-based sources: coal, oil and gas. What will it cost to replace these with clean energy sources?

A: The world will need to substantially decarbonize the energy system by the middle of this century and reach net zero emissions sometime around 2070 in order to have a high chance of keeping average global temperatures from rising more than 2 degrees centigrade. Even more rapid decarbonization would be needed to achieve the 1.5-degree centigrade limit also set in the Paris Climate Agreement. Since fossil fuel combustion is a major source of smog and air pollution, the shift to low-carbon and zero-carbon energy sources will also help to clean up the air, especially in the highly polluted cities of Asia. In many highly polluted places today, the enormous gains in health and productivity would cover much or all of the incremental costs of moving to low-carbon energy, not to mention the decisive benefits in reducing or stopping human-induced climate change. The incremental costs of decarbonization are likely to be under 1% of annual national income for typical economies, and for some economies much less than that. The benefits of stopping climate change and improving public health would be vastly greater.

“In many highly polluted places today, the enormous gains in health and productivity would cover much or all of the incremental costs of moving to low-carbon energy.”

Q: According to the International Energy Agency photovoltaic and solar-thermal plants may meet most of the world's demand for electricity by 2060. How can the global economy become nearly 100% carbon free by 2070?

A: Reaching zero net emissions by 2070 will rely on three main pillars: more efficient use of energy (more economic services per unit of energy), zero-carbon electricity, and electrification of vehicles and buildings that use clean sources of electricity. Zero or low-carbon power can be achieved in several ways: renewables (wind and solar), geothermal, hydroelectric power, nuclear energy, carbon capture and storage, ocean energy, and advanced biofuels. Countries must now consider their choices among the low-carbon and zero-carbon options, taking into account what is and can be made available in each country in terms of alternative forms of low-carbon or zero-carbon primary energy sources. The Deep Decarbonization Pathways Project, for example, has explored a range of solutions for 16 of the countries that emit the most carbon.

Q: Are the costs of switching to renewable energy sources ever balanced against the cost to health services if this switch is not made in the next few decades?

A: Not always, but there is a growing recognition that they should be. Vast numbers of lives will be saved from respiratory and other diseases caused by particulates and other forms of air pollution. The health benefits from decarbonization will not only raise life expectancy by several years in some highly polluted cities today, but will also give a direct boost to productivity and economic output. In preparing their long-term low greenhouse gas emission development strategies known as “LEDS” that are called for in the Paris Agreement (article IV, paragraph 19), countries should measure the health gains they are likely to achieve by making the switch from fossil fuels and incorporate gains into their net benefit calculations.

Q: A recent International Monetary Fund study estimated that global energy subsidies in 2015 amounted to about US$ 5.3 trillion, or 6.5% of global gross domestic product (GDP). Half of this value is due to the fact that the adverse health effects of air pollution – respiratory diseases and cardiovascular disease – are not being reflected in the price of fossil fuels. Why should the health effects of fossil fuels be included in the price of these fuels?

A: Markets are efficient only when market prices reflect the true social costs and benefits of goods and services. Fossil fuels, for example, are under-priced since their market prices do not include the social costs of climate change and air pollution, and since the market prices of the fossil fuels are often reduced in national markets through direct financial subsidies to consumers or large industrial users.

Q: So how can governments make the energy sector reflect the true social cost of fossil-fuel energy subsidies in future?

A: One key part of the strategy of decarbonization – one that governments accept is needed – is to eliminate direct financial subsidies to consumers and industrial users, and to impose an additional tax on fossil fuels to reflect the costs of the carbon emissions they produce in terms of climate change (and the damages thereby created) and air pollution with the attendant costs in mortality and adverse health effects. Carbon taxes have been implemented in several countries, for example, Denmark, Finland, Norway, Sweden and Switzerland.

Q: Some energy economists suggest that the renewable energy sector may not be as profitable as earlier predicted and may need subsidies to remain sustainable. How can governments and industry ensure that clean energy provision is sustainable and viable? 

A: To ensure the shift from fossil fuels to renewables and other low-carbon energy sources (including hydroelectric, geothermal, and nuclear energy, depending on the region of the world), we will need a comprehensive set of policies including carbon pricing, regulation, land-use decisions, public-sector procurement, and public support for research and development. There is no single policy instrument that will bring about timely and comprehensive decarbonization. Many policy tools deployed simultaneously will be needed, including carbon pricing. And there are many possible kinds of carbon pricing, including a direct tax on emissions, a tradable permit system (for the total amount of carbon that can be emitted), or some kind of subsidy for low-carbon energy.

Q: Two major economies, China and the United States of America, are now taking action to reduce their carbon emissions. What are the economic consequences of such action in the short- and long-term? 

A: Both China and the United States (as well as other countries, including Canada) have announced their intention to undertake detailed long-term analysis – in the sustainable development community we call them “deep decarbonization pathways” – in order to answer the questions about the strategies and costs of long-term decarbonization. Under the Paris Agreement, all countries are obliged to prepare long-term, low greenhouse-gas emission development strategies and to present these long-term plans to the global community by 2020 at the latest. The early evidence suggests that deep decarbonization is fully feasible, and will cost around 1% or less of national income per year until 2050, with social benefits in health, increased quality of life, and reduced climate change, that amount to much more than 1% of national income per year.

“It is probably sound to allow the poorest countries, notably in Africa, to continue to use their local fossil fuel.”

Q: Many wealthy, industrialized countries accept that carbon emissions must be reduced but what about emerging economies that are still growing? The belief has been that they need fossil fuels for rapid growth. Is this true and have the economics been weighed up against the dire health effects of such an approach?

A: We still need to study more the fair allocation of decarbonization across countries. Eventually, all countries will need to decarbonize comprehensively, but it is probably sound to allow the poorest countries, notably in Africa, to continue to use their local fossil fuel resources for longer than is the case in the middle-income and high-income countries. These very poor countries contribute only a very small part of global emissions, but represent a large share of the global poor. Moreover, the richer countries of course have a vastly higher share of the cumulative historical emissions to date, and therefore a vastly higher responsibility for the damages that are already underway.

